# Traffic noise causes physiological stress and impairs breeding migration behaviour in frogs

**DOI:** 10.1093/conphys/cou032

**Published:** 2014-08-16

**Authors:** Jennifer B. Tennessen, Susan E. Parks, Tracy Langkilde

**Affiliations:** 1 Department of Biology, Intercollege Graduate Degree Program in Ecology, Center for Brain, Behavior and Cognition, The Pennsylvania State University, 208 Mueller Laboratory, University Park, PA 16802, USA; 2 Department of Biology, Syracuse University, 258 Life Sciences Complex, 107 College Place, Syracuse, NY 13244, USA

**Keywords:** Corticosterone, frog, mate attraction, noise, stress

## Abstract

Human-generated noise has profoundly changed natural soundscapes in aquatic and terrestrial ecosystems, imposing novel pressures on ecological processes. Despite interest in identifying the ecological consequences of these altered soundscapes, little is known about the sublethal impacts on wildlife population health and individual fitness. We present evidence that noise induces a physiological stress response in an amphibian and impairs mate attraction in the natural environment. Traffic noise increased levels of a stress-relevant glucocorticoid hormone (corticosterone) in female wood frogs (*Lithobates sylvaticus*) and impaired female travel towards a male breeding chorus in the field, providing insight into the sublethal consequences of acoustic habitat loss. Given that prolonged elevated levels of corticosterone can have deleterious consequences on survival and reproduction and that impaired mate attraction can impact population persistence, our results suggest a novel pathway by which human activities may be imposing population-level impacts on globally declining amphibians.

## Abstract (precis)

Noise from human activities is increasing globally. We provide evidence that traffic noise increases glucocorticoid concentrations and impairs reproductive behavior in frogs. Since prolonged stress can compromise health, survival and reproduction, and because impaired reproductive behavior can reduce mating opportunities, these results suggest noise may contribute to amphibian declines.

## Introduction

Noise generated by human activities permeates habitats throughout most of the world (Barber *et al.*, 2010) and is predicted to increase in distribution and intensity with increasing human population growth (Babisch *et al.*, 2005). Noise from road, rail and air traffic activities is audible above baseline ambient sound in most counties in the continental USA (Barber *et al.*, 2010). Anthropogenic noise is known to alter human behaviour and cause stress-related diseases (Babisch *et al.*, 2005; Öhrström *et al.*, 2006; Goines and Hagler, 2007), but far less is known about its impacts on wildlife populations. Many species rely on sound for critical fitness-related functions, including mate attraction, territory defense, predator detection and foraging (Bradbury and Vehrencamp, 2011). These activities are adapted to maximize signal transmission and detection within specific acoustic environments, characterized by combinations of spectral and temporal acoustic properties (the ‘acoustic adaptation hypothesis’; Morton, 1975). Noise created as a byproduct of human activities represents a novel pressure on acoustic habitats by altering the acoustic properties of the environments in which species' communication systems evolved.

Research on the impacts of anthropogenic noise on wildlife has focused primarily on identifying short-term behavioural responses and has revealed that many species modify components of their acoustic signals in order to maintain successful communication in noisy environments (e.g. Miller *et al.*, 2000; Brumm and Slabbekoorn, 2005; Lengagne, 2008; Parks *et al.*, 2011). Anurans (frogs and toads), in particular, adjust their vocal behaviour in noisy environments by ceasing calling, calling faster or modifying call frequency or amplitude (e.g. Sun and Narins, 2005; Lengagne, 2008; Kaiser and Hammers, 2009; Parris *et al.*, 2009; Cunnington and Fahrig, 2010; Vargas-Salinas and Amézquita, 2013; Penna and Zuniga, 2014). While these short-term behavioural responses presumably improve signal detection by receivers (Cunnington and Fahrig, 2013) and thus may contribute to maintaining successful communication in noisy habitats (Slabbekoorn, 2013), the sublethal effects of noise, including physiological stress and impaired reproduction, remain poorly understood (Kight and Swaddle, 2011).

Anurans are an important group in which to examine the behavioural and physiological consequences of noise owing to the potential conservation implications of noise impacts on this group. Many anurans breed in water bodies created by roads (e.g. drainage ditches, retention ponds, borrow pits). Consequently, noise exposure is common to many anuran species. Sound plays a fundamental role in individual fitness in most anurans through acoustic breeding displays, mate attraction, territory defense and predator detection (Gerhardt and Huber, 2002); therefore, changes in the acoustic environment due to anthropogenic noise may impact anuran population dynamics (Kaiser *et al.*, 2011). Female anurans exhibit phonotaxis towards male choruses (Gerhardt and Huber, 2002; Bee and Swanson, 2007), and increases in the detection threshold of a signal due to noise may impair or impede an individual's ability to detect and respond to biologically critical information (Barber *et al.*, 2010), which could affect mate attraction (Bee and Swanson, 2007). Determining the impacts of associated traffic noise is pertinent to understanding factors that may be contributing to widespread declines in amphibian populations.

In addition to experiencing impaired signal detection, anurans may suffer physiological stress in response to anthropogenic noise, as has been shown in response to other novel threats, including pollution and habitat fragmentation (Relyea and Mills, 2001; Janin *et al.*, 2011), resulting in the release of corticosterone. Corticosterone is the primary glucocorticoid hormone in amphibians and forms part of the highly conserved vertebrate physiological stress response (Wingfield *et al.*, 1998; Sapolsky *et al.*, 2000; Romero, 2004). Short-duration elevations of plasma corticosterone concentrations help an organism to respond adaptively to stressors by facilitating the mobilization of energy stores, suppressing unnecessary activities and priming the response to future stressors (Sapolsky *et al.*, 2000; Romero, 2004). Chronically elevated corticosterone concentrations, however, can have deleterious effects on survival, reproduction, growth and immune function, due largely to a reallocation of energy away from non-critical functions (Sapolsky *et al.*, 2000). Thus, if traffic noise triggers elevations in plasma corticosterone concentrations in anurans, the population-level consequences may be substantial. Assessment of the physiological and behavioural impacts of anthropogenic noise on anurans will provide important insights into individual and population-level effects of this growing ecological pressure.

**Figure 1:**
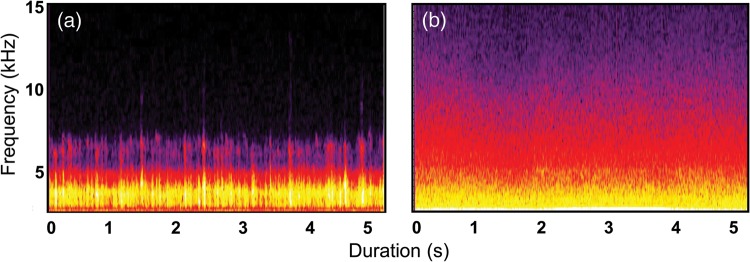
Wood frog breeding choruses overlap in frequency with traffic noise. Spectrograms illustrating male wood frog chorus (**a**) and road traffic noise (**b**) recorded in Centre County (PA, USA). Lighter shading indicates greater acoustic energy at a particular time and frequency. The frequency characteristics of traffic noise overlap those of wood frog chorus, with the greatest energy of both occurring between 0 and 3 kHz, suggesting that noise may interfere with conspecific detection of wood frog breeding choruses.

We used female wood frogs (*Lithobates sylvaticus*) to determine whether traffic noise (i) impacts female breeding migration behaviour in the natural environment and (ii) causes an elevation in corticosterone, an indicator of physiological stress. Wood frogs are primarily terrestrial, with an explosive pond-breeding period governed by male–male competition, which lasts only a few days each year following the spring thaw (Berven, 1981). Males migrating to woodland ponds begin chorusing immediately upon entry, and females arrive soon after (Wells, 1977). Evidence suggests that wood frogs may use chorus sounds to locate these ephemeral breeding aggregations (Bee, 2007). Given that wood frog habitat overlaps a vast and increasing network of roads characterized by intermittent to continuous traffic noise (Barber *et al.*, 2011), road traffic noise, which spans the frequency range of wood frog calls (Fig. 1), may therefore interfere with breeding migration and elicit a physiological stress response.

We predicted that traffic noise impacts would be manifested in the following two ways: (i) as female anurans exhibit phonotaxis towards male choruses (Gerhardt and Huber, 2002; Bee and Swanson, 2007), signal interference should delay their breeding migration; and (ii) acoustic habitat loss from traffic noise should elevate circulating corticosterone concentrations, as occurs in response to loss of physical habitat (Janin *et al.*, 2011). We conducted two acoustic playback experiments to determine whether noise (i) disrupts female travel towards a breeding chorus, and (ii) increases baseline plasma corticosterone concentrations in female wood frogs.

## Materials and methods

### Field collection

Gravid female wood frogs [*n* = 66; 43.3–62.8 mm snout–urostyle length (SUL), gravidity determined by visual inspection of body shape] were captured from quiet sites (2 km from the nearest high-traffic road) within the Pennsylvania State Game Lands in Scotia, Pennsylvania (coordinates: 40.780 N, 78.007 W), during migration from over-wintering sites to vernal ponds, using drift fence and pitfall trap sampling. Females were placed in a 53-l plastic tub with a thin layer of damp soil and leaves for cover, and transported 1 km to the field experiment site, where they were held in these conditions for one to five nights until they were used in the field reproductive behaviour experiment (as per Schwartz *et al.*, 2000; Bee, 2007; Bee and Swanson, 2007; Gerhardt, 2008).

### Field reproductive behaviour experiment

**Figure 2:**
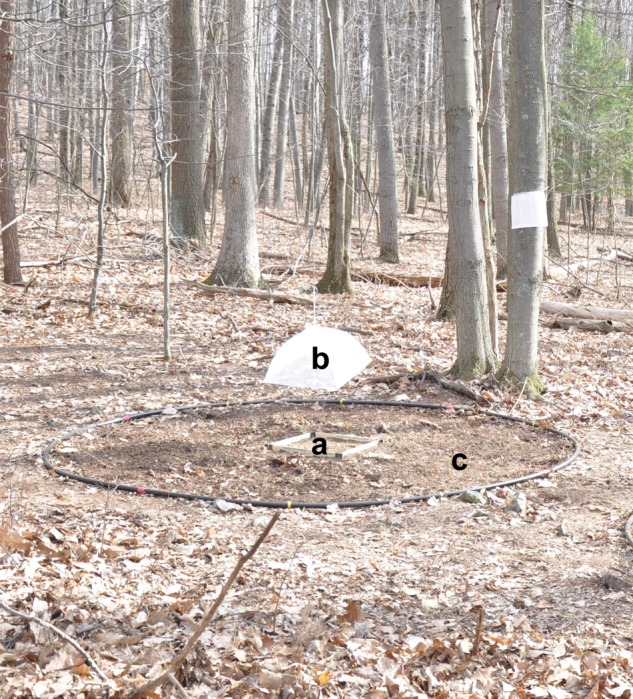
A photograph of the experimental set-up used for the field reproductive behaviour experiment. During the initial playback, an individual frog was placed within a holding area (a), beneath a mesh dome (b; partly suspended in this image), inside a 3-m-diameter arena (c). The dome was then lifted by a remotely operated pulley system, and the frog was allowed to travel freely for 5 min or until it cleared the arena, whichever occurred first.

We randomly assigned gravid female wood frogs to receive one of four combinations of male wood frog chorus and synthetic traffic noise, such that noise represented 0, 28, 97 or 100% of the total signal energy, or a silence control (see ‘*Design of playback stimuli*’, below). For each trial, we placed a female on natural substrate (soil that was raked to remove leaf litter) in the centre of a 3-m-diameter arena underneath a mesh dome attached to an overhead remotely operated pulley system (Fig. 2). The observer (J.B.T.) then retreated behind a portable shelter for the duration of the trial so that her presence would not disturb the frog. In order to control for directional biases, we alternated trials between two arenas with mirror-image orientations. Following a 5 min acclimation period, we played the appropriate acoustic treatment to the female for 3 min using an iPod Nano (Apple, Inc., Cupertino, CA, USA) connected to an Amp10 Exterior Receiver Amplifier (TIC Corporation, City of Industry, CA, USA) that powered two GS5 Mini Omnidirectional Speakers (TIC Corporation), placed 1.5 m from the arenas (system frequency response, 55–16 000 Hz). After the 3 min stimulus playback, we remotely raised the dome, allowing the female to travel freely while the acoustic treatment continued for 5 min (±5%) or until the frog cleared the boundary of the arena, whichever occurred first, and we recorded latency to clear the arena. We then measured the female's SUL (in millimetres) using electronic digital callipers and mass (in grams) using a spring scale, and these were used to calculate body condition [residuals of a regression of ln(mass) vs. ln(SUL)].

### Stress physiology experiment

Following the field behaviour experiment (see previous subsection), gravid female wood frogs (*n* = 54) were allowed to mate in outdoor enclosures. Females were then transported to the laboratory, housed individually in 18-l plastic containers, fed crickets every 2 days and acclimated in constant laboratory conditions (18°C, 12 h–12 h light–dark cycles) for 2.5 weeks prior to commencing the experiment. Following acclimation, females were exposed to one of three different acoustic treatments for at least 12 h, followed by immediate blood collection. We used a stratified random design to assign each of three playback treatments to one of three rooms on each of three nights. Playback treatments consisted of silence, male wood frog chorus or chorus + high traffic noise (see ‘*Design of playback stimuli*’). Eighteen females, each in individual enclosures, were randomly assigned to each playback treatment (six females in each of three playback treatment groups per night, for three nights). Each enclosure containing a female was randomly placed on a rack against a wall in each playback room (six per room; vertical and horizontal surfaces were partly covered with sound-absorbing materials to minimize reverberation). The acoustic treatment was delivered with a MacBook Pro laptop computer (Apple, Inc.) to an Amp10 Exterior Receiver Amplifier (TIC Corporation) or Dual XPA2100 (Dual Electronics Corporation, Heathrow, FL, USA) that drove a GS5 Mini Omni-Directional Speaker (TIC Corporation; system frequency response, 55–16 000 Hz). The speaker was placed on the floor in each room and adjusted such that the source level was 87 dBA re 20 µPa at 1 m from the speaker, using a hand-held digital sound level meter, for both the chorus and chorus + noise treatments, representative of conditions we recorded in the field. For individuals receiving the silence control, the playback equipment was set up identically to that for individuals receiving the chorus or chorus + noise treatments, but no sounds were played. Broadcasting of acoustic stimuli commenced shortly after the start of the 12 h dark period, and stimuli were delivered continuously overnight, during which time the individuals were left undisturbed.

Twelve hours after commencing the playback, and while the playback was still occurring, we drew up to 100 µl of blood by cardiac puncture using a new heparinized needle for each individual. We were able to obtain sufficient blood from three to six frogs per group. Blood samples were stored on ice until all frogs were sampled, immediately centrifuged to separate red blood cells from plasma, and plasma was stored at −20°C until assayed. We determined baseline plasma corticosterone concentration using enzyme immunoassay [Corticosterone High Sensitivity EIA Kits, Immunodiagnostic Systems (IDS) Inc., Scottsdale, AZ, USA; validated for this species]. Sample concentrations were determined from a standard curve (*r*^2^ = 0.9783), calculated using a calibrator of known concentration and a five-increment serial dilution following instructions provided in the kit. Plasma was initially diluted 80% with assay buffer (10 µl plasma + 40 µl buffer) so that samples fell within the detectable range of the standard curve. All samples were run on a single plate, with two controls, and assayed in duplicate. Intra-assay coefficient of variation was 5.18%, calculated from values of the provided controls run in quadruplicate.

Following the playback experiment, females were measured for mass and then released in the field at their point of capture (body condition was calculated using SUL from the field experiment, because this was unlikely to have changed over this short time period).

### Design of playback stimuli

Acoustic recordings of a male wood frog chorus were collected during the afternoon of 22 March 2011, from a height of 1 m above the ground, adjacent to a vernal pond in the Pennsylvania State Game Lands in Scotia (PA, USA), using a Marantz PMD 620 Professional Handheld Digital Audio Recorder (Marantz America, LLC, Mahwah, NJ, USA; frequency response, 20–20 000 Hz), sampling at 44.1 kHz. To create synthetic traffic noise, recordings were made during the evening peak traffic period along US Route 322 adjacent to the Penn State campus in University Park (PA, USA) on 28 February 2012, using a G.R.A.S. 40AE pre-polarized microphone (G.R.A.S. Sound & Vibration A/S, Holte, Denmark) connected to a G.R.A.S. 26CA preamplifier and a Marantz PMD 660 Portable Compact Flash Recorder (Marantz America, LLC; system frequency response, 20–20 000 Hz), sampling at 44.1 kHz. A white noise signal was digitally generated with a sampling rate of 48 000 Hz, using a custom Matlab script in Matlab R2007a (The Mathworks, Inc., Natick, MA, USA). This white noise signal was digitally filtered using Adobe Audition CS5.5 (Adobe Systems, Inc., San Jose, CA, USA) and a custom, traffic-shaped filter created using the average frequency and amplitude envelopes of the recorded traffic noise. Acoustic playback stimuli were created from stereo combinations of 2 min periods of silence, chorus or synthetic traffic noise using Adobe Audition CS5.5, and repeated five times to create signals with 10 min durations.

To create stimuli for the reproductive behaviour experiment, we constructed stereo files representing no, low, high and only traffic noise treatments, by combining mono files of chorus and noise to which we had initially applied an intensity-equalizing function and then dropped the amplitude for the noise files accordingly using Adobe Audition CS5.5. We computed mean-square averages of the total energy in each channel of the stereo files using a custom script in Matlab R2007a and quantified the percentage of noise present. Noise composed 0, 28, 97 and 100% of the acoustic energy in the playback stimuli, respectively. Given that *L. sylvaticus* hearing is poorly understood, the ‘noise only’ treatment was designed to create the scenario where masking noise prevents any chorus detection. The silence treatment consisted of a silent stereo audio file (zero amplitude), and was played to frogs using the same protocol as the other acoustic treatments. For the stress physiology experiment, we used the silence, 0 and 97% noise present stimuli from the reproductive behaviour experiment for the silence, chorus, chorus + noise stimuli, respectively, and set these 10 min files to loop automatically for the 12 h duration of the experiment.

### Statistical analysis

To evaluate the effects of acoustic treatment on female likelihood to clear the arena, we fitted Cox proportional hazards survival analysis models with right censoring, with acoustic treatment as a factor, using the ‘survival’ package (Therneau and Lumley, 2011) in R version 2.13.1 (R Development Core Team, 2011, R Foundation for Statistical Computing, Vienna, Austria). To determine whether acoustic treatment affected the time to leave the arena for the subset of individuals that left, we used a mixed-model ANOVA (in JMP 10; SAS Institute Inc., Cary, NC, USA), with acoustic treatment as a factor.

To evaluate the effects of acoustic treatment on plasma corticosterone concentrations, we used a mixed model (in JMP 10), with acoustic treatment as a fixed factor and group[acoustic treatment] as a random factor. We ran Tukey's HSD *post hoc* tests to compare mean corticosterone concentrations between treatment groups. A significance level of α = 0.05 was used for all analyses.

Snout–urostyle length and corticosterone concentrations were natural log transformed to meet assumptions of parametric analyses. Snout–urostyle length and body condition [residuals of the regression of ln(mass) vs. ln(SUL)] were originally included as covariates in the analysis of the behavioural trial data, but did not significantly explain variation in the likelihood to clear the arena (*P* > 0.321) or in female time to leave the arena (*P* > 0.356). Likewise, corticosterone concentration was not affected by blood collection duration, playback duration, SUL or body condition (*P* > 0.396). These covariates were thus omitted from the final model.

## Results

### Reproductive behaviour experiment

**Figure 3:**
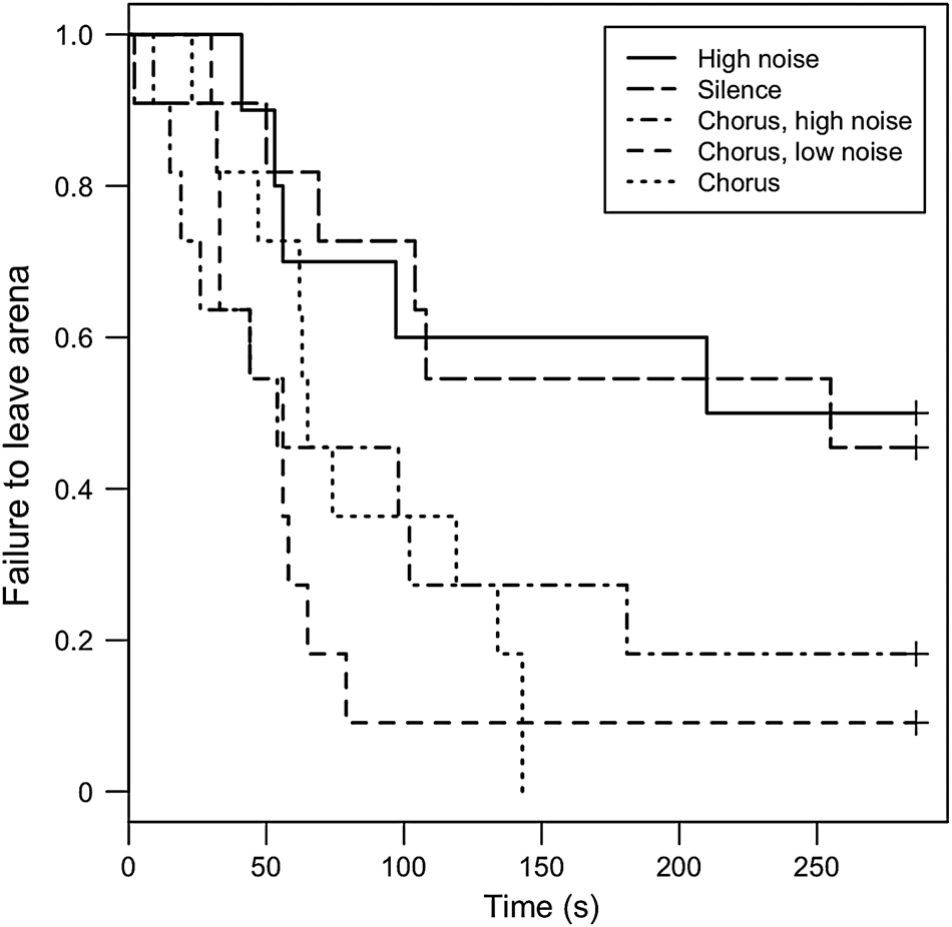
Absence of chorus impairs wood frog mobility. Survivorship curves representing failure of female wood frogs to leave the experimental arena when receiving chorus (dotted line), chorus + low traffic noise (short-dashed line), chorus + high traffic noise (dashed and dotted line), silence (long-dashed line), and high traffic noise (continuous line) acoustic treatments (*n* = 11, 11, 11, 11 and 10, respectively). Plus (+) symbols at the ends of the curves indicate treatment groups in which not all individuals left the arena within the trial period.

**Figure 4:**
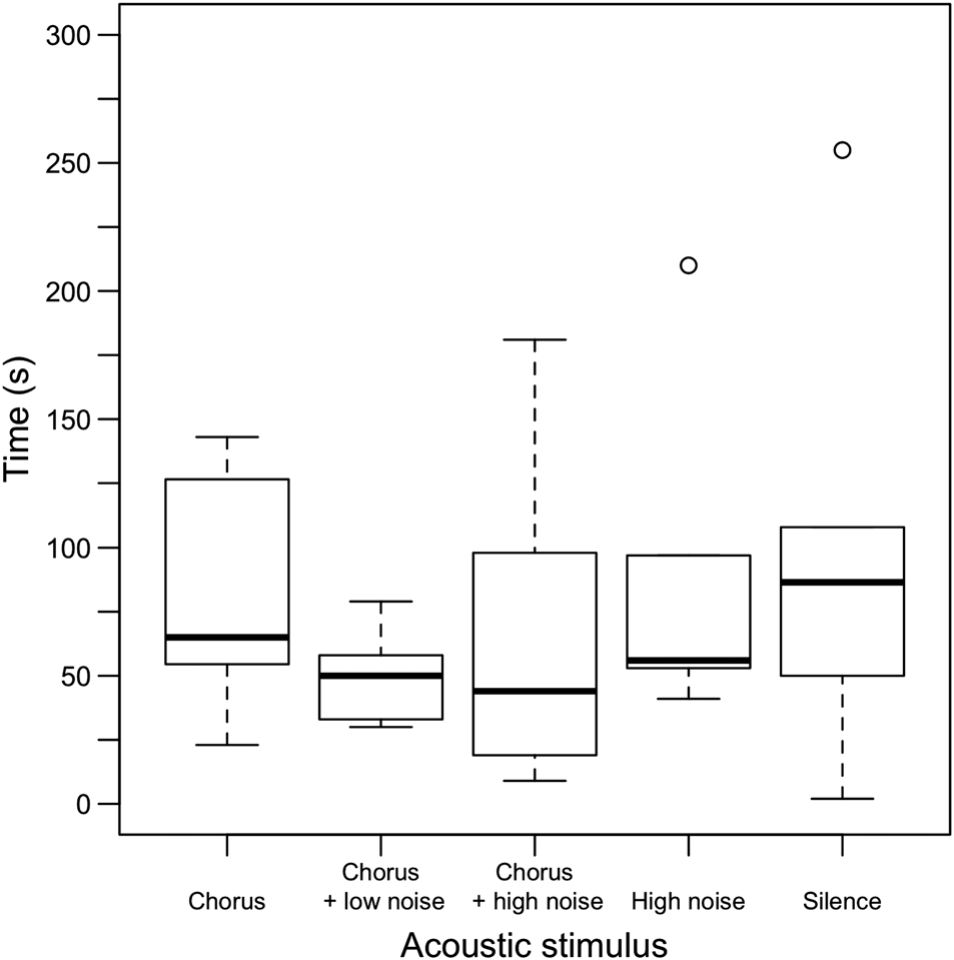
Acoustic stimulus had no effect on time to leave the arena for the subset of individuals that left. Sample sizes for chorus, chorus + low noise, chorus + high noise, high noise and silence are *n* = 11, 10, 9, 5 and 6, respectively. Boxes represent interquartile ranges, horizontal lines represent medians, and vertical dashed lines represent adjacent values not greater than 1.5 times the height of the box.

Acoustic treatment significantly affected the movement of gravid female wood frogs in the field (Λ_4_ = 12.58, *P* = 0.014; Fig. 3). *Post hoc* analyses revealed that significantly fewer females cleared a 3-m-diameter arena in their natural forest habitat when chorus was absent (chorus vs. noise, *n* = 21, *P* = 0.033; and chorus vs. silence, *n* = 22, *P* = 0.034), and there was a similar but non-significant trend for the chorus vs. chorus + low noise and chorus vs. chorus + high noise groups (*n* = 22, *P* = 0.518 and *n* = 22, *P* = 0.760, respectively). There was no effect of acoustic treatment on time to clear the arena for the subset of individuals that left the arena within the given trial period (*F*_4,36_ = 0.740, *P* = 0.571; Fig. 4).

### Stress physiology experiment

**Figure 5:**
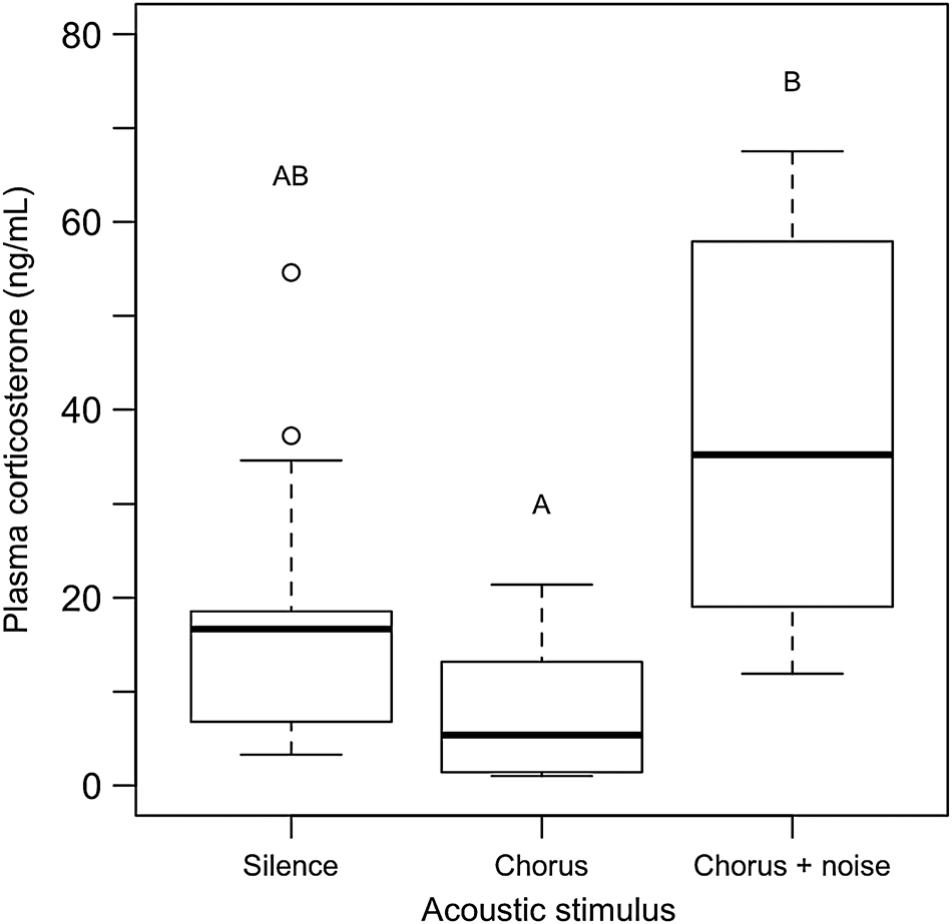
Traffic noise causes physiological stress in wood frogs. Concentrations of plasma corticosterone in female wood frogs exposed to one of three acoustic treatments: silence, male wood frog chorus or chorus + high traffic noise (*n* = 13, 12 and 13, respectively). Boxes not connected by the same letter are significantly different. Boxes represent interquartile ranges, horizontal lines represent medians, and vertical dashed lines represent adjacent values not greater than 1.5 times the height of the box.

Acoustic treatment had a significant effect on corticosterone concentration (*F*_2,6_ = 12.112, *P* = 0.008). Tukey's HSD *post hoc* tests revealed that plasma concentrations of individuals exposed to chorus + traffic noise were five times greater than those of individuals exposed to chorus alone (Fig. 5).

## Discussion

Traffic noise is widespread and increasing in intensity and distribution (Barber *et al.*, 2010). This study demonstrates that traffic noise can impact anuran reproductive behaviour in the field and provides, to our knowledge, the first evidence that traffic noise can cause physiological stress in anurans. These results offer insight into the behavioural and physiological mechanisms through which anthropogenic noise may impact populations.

Female wood frog travel in the field was impaired in the absence of chorus. In the presence of a male chorus with no traffic noise, all females travelled out of the field arena within 5 min. Fewer females cleared the field arena as the signal-to-noise ratio decreased (greater road noise, less chorus noise), with only 50% of females clearing the arena when we mimicked noise completely masking the male chorus, and 55% clearing the arena in the silence control. Increased levels of ambient noise are associated with impaired signal detection and discrimination in acoustic species (e.g. frogs and birds; Wollerman, 1999; Lohr *et al.*, 2003), and increased female response latency and decreased orientation towards a broadcasted male call in laboratory experiments (grey treefrog, *Hyla chrysoscelis*; Bee and Swanson, 2007), suggesting that reduced female movement in our trials may have been caused by chorus masking, resulting in females being unable to locate and orient towards the male calls. This hypothesis is consistent with the result that, in the absence of an audible chorus (the high noise and silence treatments), females were significantly less likely to clear the arena.

An alternative explanation is that traffic noise impaired female travel by activating an immobility stress response, potentially mediated by corticotropin-releasing factor in the brain (Carr *et al.*, 2002, 2013), linking an underlying physiological mechanism to an observed behavioural response. Tonic immobility is a common anuran behavioural and physiological response to stress (Lupo *et al.*, 1991), and the results of our laboratory playback study (that noise elevated corticosterone concentrations) and the speed at which frogs exhibit tonic immobility when stressed (Lupo *et al.*, 1991) suggest that noise-induced tonic immobility may explain the reduction in female movement in the presence of noise. All but two (83%) of the frogs that failed to leave the arena within 5 min remained stationary for the duration of the trial, further supporting this explanation. If this alternative explanation is correct, it is surprising that the silence treatment likewise caused a significant reduction in likelihood to clear the arena. It is possible that silence itself is an alerting stimulus (Dapper *et al.*, 2011) that may trigger a tonic immobility response.

In either case, high levels of traffic noise, which are experienced adjacent to interstates and other major roads, may impair reproductive behaviour because immobility, whether due to masking or stress, would presumably delay a female's arrival at breeding aggregations. Given that the timing of breeding events in seasonal environments should have evolved to maximize survival and growth of offspring (Lack, 1954), females experiencing noise-induced delays in arrival at breeding sites may therefore experience lower (or no) fitness due to availability of poor-quality mates, decreased fertilization rates or reduced offspring success. Indeed, such priority effects can impact the outcomes of competition and predation among developing offspring in pond communities (Morin, 1987).

It is important to note, however, that because the trial period lasted for 5 min, we cannot rule out the possibility that frogs may behaviourally habituate to noise over time. The frogs used in this experiment were likely to be naïve to traffic noise. It would be interesting to compare our results with tests of frogs from highway-adjacent populations to determine whether habituation or adaptation to noise has occurred, and if these frogs display a reduced or absent tonic immobility response. Additionally, studies that explore the directionality of female migration towards a variety of chorus and non-chorus signals, and how altered movement caused by traffic noise may contribute to amphibian road mortality, which is known to impact amphibian populations (e.g. Fahrig *et al.*, 1995; Trombulak and Frissell, 2000; Hels and Buchwald, 2001; Mazerolle, 2004; Eigenbrod *et al.*, 2008), may be valuable avenues for future research.

In addition to impaired reproductive behaviour, traffic noise significantly increased female plasma corticosterone concentrations. Similar elevations in corticosterone concentrations have been documented in anuran responses to habitat loss (Janin *et al.*, 2011) and pollution (Relyea and Mills, 2001), lending support to our findings of this physiological response to loss of acoustic habitat due to traffic noise. The physiological costs of noise-induced stress may be substantial. While glucocorticoids help animals respond adaptively to stressors in the short term (Sapolsky *et al.*, 2000), chronically elevated glucocorticoid levels can have deleterious consequences, including suppressed immune function and reproduction and reduced survival (e.g. Romero and Wikelski, 2001; Martin *et al.*, 2005; Pride, 2005; Ouyang *et al.*, 2011; but see Bonier *et al.*, 2009). Our results, combined with recent studies identifying linkages between increased anthropogenic noise and elevated glucocorticoid levels in fish, birds and cetaceans (Smith *et al.*, 2004; Anderson *et al.*, 2011; Hayward *et al.*, 2011; Rolland *et al.*, 2012), suggest that the physiological consequences of noise span vertebrate taxa. Future studies that determine whether individuals from noisy environments are chronically stressed or able to acclimate or become adapted to noise stressors (and either cease responding or mount a reduced stress response), as well as studies that link noise-induced increases in glucocorticoids to other measures of physiological health and behaviour, would provide important contributions to this field. Anuran populations are declining globally (Houlahan *et al.*, 2000), and several abiotic and biotic factors have been implicated (Blaustein and Kiesecker, 2002). Habitat loss due to roads, in particular, has been associated with amphibian population declines, including mortality due to contamination with chemical runoff (Sanzo and Hecnar, 2006; Karraker *et al.*, 2008; Brady, 2012) and vehicle-associated mortality (Hels and Buchwald, 2001; Eigenbrod *et al.*, 2008). Anthropogenic noise creates another form of habitat loss (Barber *et al.*, 2010), and anurans may be particularly susceptible to such acoustic habitat degradation (Bee and Swanson, 2007). Our results suggest that the sublethal impacts of anthropogenic noise on anurans may be under-appreciated and may contribute to the growing evidence, across taxa, of the need to address the increasing acoustic degradation of the natural environment that is a consequence of human population growth and innovation.

## Funding

This work was supported by The Pennsylvania State University [Academic Computing Fellowship to J.B.T., and the Applied Research Laboratory] and by the National Science Foundation [IOS-1051367 to T.L.].
